# Men’s roles in care seeking for maternal and newborn health: a qualitative study applying the three delays model to male involvement in Morogoro Region, Tanzania

**DOI:** 10.1186/s12884-019-2439-8

**Published:** 2019-08-13

**Authors:** Jesse A. Greenspan, Joy J. Chebet, Rose Mpembeni, Idda Mosha, Maurus Mpunga, Peter J. Winch, Japhet Killewo, Abdullah H. Baqui, Shannon A. McMahon

**Affiliations:** 1 Department of International Health, Johns Hopkins Bloomberg School of Public Health, 615 North Wolfe Street, Baltimore, MD USA; 2 Partners In Health, 800 Boylston Street, Suite 300, Boston, MA USA; 3 Department of Health Promotion Sciences, Mel and Enid Zuckerman College of Public Health, University of Arizona, Tucson, AZ USA; 4 Department of Epidemiology and Biostatistics, School of Public Health and Social Sciences, Muhimbili University of Health and Allied Sciences, P.O. Box 65015, Dar-Es-Salaam, Tanzania; 5 Department of Behavioural Sciences, School of Public Health and Social Sciences, Muhimbili University of Health and Allied Sciences, P.O. Box 65015, Dar-Es-Salaam, Tanzania; 6 Department of Labour Studies, Institute of Social Work, P.O. Box 3375, Dar-es-Salaam, Tanzania; 7 Institute of Public Health, Ruprecht-Karls-Universität, Heidelberg, Germany

**Keywords:** Male involvement, Care seeking, Gender, Maternal health, Newborn health, Healthcare financing, Tanzania

## Abstract

**Background:**

Increasing the utilization of facility-based care for women and newborns in low-resource settings can reduce maternal and newborn morbidity and mortality. Men influence whether women and newborns receive care because they often control financial resources and household decisions. This influence can have negative effects if men misjudge or ignore danger signs or are unwilling or unable to pay for care. Men can also positively affect their families’ health by helping plan for delivery, supplementing women’s knowledge about danger signs, and supporting the use of facility-based care. Because of these positive implications, researchers have called for increased male involvement in maternal and newborn health. However, data gathered directly from men to inform programs are lacking.

**Methods:**

This study draws on in-depth interviews with 27 men in Morogoro Region, Tanzania whose partners delivered in the previous 14 months. Debriefings took place throughout data collection. Interview transcripts were analyzed inductively to identify relevant themes and devise an analysis questionnaire, subsequently applied deductively to all transcripts.

**Results:**

Study findings add a partner-focused dimension to the three delays model of maternal care seeking. Men in the study often, though not universally, described facilitating access to care for women and newborns at each point along this care-seeking continuum (deciding to seek care, reaching a facility, and receiving care). Specifically, men reported taking ownership of their role as decision makers and described themselves as supportive of facility-based care. Men described arranging transport and accompanying their partners to facilities, especially for non-routine care. Men also discussed purchasing supplies and medications, acting as patient advocates, and registering complaints about health services. In addition, men described barriers to their involvement including a lack of knowledge, the need to focus on income-generating activities, the cost of care, and policies limiting male involvement at facilities.

**Conclusion:**

Men can leverage their influence over household resources and decision making to facilitate care seeking and navigate challenges accessing care for women and newborns. Examining these findings from men and understanding the barriers they face can help inform interventions that encourage men to be positively and proactively involved in maternal and newborn health.

## Introduction

Increasing women’s and children’s encounters with effective health services can greatly reduce maternal and child morbidity and mortality in low-resource settings [1]. According to the three delays model, a seminal framework describing maternal care seeking for obstetric emergencies, there are three types of barriers that can hinder care seeking: 1) a delay in deciding to seek care (affected by perceptions of illness severity or cause, the status of women, distance, ease of transportation, cost, and the quality of care available); 2) a delay in reaching care (affected by distance, road conditions, travel time, and transportation cost or availability); and 3) a delay in receiving care once at the facility (affected by shortages of well-trained staff, drugs, supplies, and equipment, and the ability of the referral system to respond to patients’ needs) [2]. Men are often implicated in delays seeking emergency or other types of care given that decisions related to seeking care outside the home are often based on the opinion of men and male household heads [3–5]. This level of decision-making authority means that men’s incorrect judgment regarding danger signs, when to depart for a facility, or the need for a facility delivery or referral can delay care seeking and endanger mothers and newborns [6–8].

However, as Davis, Luchters and Holmes described in their 2013 review, engaging men in maternal and newborn health and providing them with knowledge for informed decision-making can contribute positively to the health of their families by decreasing maternal workload, increasing emotional support for women during pregnancy, improving birth preparedness, increasing use of health services during the postnatal period, and improving couple communication [5]. Furthermore, male accompaniment during antenatal care specifically can positively affect facility-based deliveries and skilled attendance at birth, women’s knowledge about danger signs, and utilization of postnatal services [6, 9]. Male participation during pregnancy and after childbirth can also lead to a decreased likelihood of maternal postpartum depression [9]. The positive impact of male involvement is not unique to low-resource settings. In high-resource settings such as the United States, Israel, Great Britain, and Sweden, male involvement has been shown to positively affect children’s cognitive development [10–12].

Men who want to be positively involved in maternal and newborn health can face substantial barriers related to local expectations of male roles. A number of terms for these expectations are found in the literature, including gender ideology [13], socialization ideology [14] gender norms, and male role norms [15], and can manifest in numerous ways. In Nepal [16, 17] and Tanzania [18], for example, men reported experiencing social stigma against male involvement. In Malawi, men expressed feeling ignored by facility staff [19]. Men also described encountering actual or perceived policies restricting their movement and presence where women’s health services are delivered in Nepal [17], Kenya [20], Rwanda [21], and Ghana [8]. Encountering barriers that limit men’s interactions with their partners and providers can cause men to feel ineffective and uninformed [22, 23].

The global public health community has recognized the importance of male involvement in maternal and newborn health for decades. Among the most explicit calls for male involvement was a mandate issued at the 1994 International Conference on Population and Development, which had a stated objective “to encourage and enable men to take responsibility for their sexual and reproductive behaviour and their social and family roles” [24]. More recently, in its 2015 recommendations on interventions for maternal and newborn health, the World Health Organization called specifically for interventions that “promote the positive role that men can play as partners and fathers” [25].

Despite evidence and calls for action, implementers have struggled to enact programs to increase male involvement due, in large part, to perceptions and implications of gender norms [5, 26]. Implementation challenges are also linked to a lack of research directly involving men on the prenatal, labor, delivery, and postpartum periods [20]. Instead, research involving men has focused on HIV prevention and treatment [27–29] and family planning [30–33]. In addition, studies often rely primarily on women’s reports of assumed male perspectives, although relying on women to serve as proxies for their husbands can be misleading; women do not always accurately report their husbands’ opinions about health-related topics [33–35] and husband-wife pairs may report divergent accounts of the same events [7].

This study draws on interviews with men in Morogoro Region, Tanzania to describe their role in maternal and newborn health care seeking. Data analysis revealed insights from men that align with the three delays model, which has been applied extensively in the maternal health literature, but has received less attention in literature focusing on male perspectives [36]. We present our findings according to this framework, adding a male-partner perspective on how men can contribute to overcoming each of the delays, and the barriers they face in doing so. In our discussion, we situate our findings relative to the literature on male participation in care seeking, focusing on men’s roles as decision maker, financial provider, and patient advocate. Lastly, drawing from respondent recommendations and conversations in the literature on gender inequity, we provide insights on how to bolster male involvement in maternal and newborn health services.

## Methods

### Study setting

The study took place in five districts of Morogoro Region: Gairo (at time of study, this district was part of Kilosa district), Kilosa, Morogoro Rural, Mvomero, and Ulanga districts. This setting is marked by male-dominated household decision making and elevated maternal mortality (see Table 1). A comparison of Tanzania’s 2010 DHS data and 2015/2016 DHS data suggests that women’s involvement in decision making has improved, although 27.5% of men remain the primary decision makers related to women’s health care, and the percentage of women reporting challenges accessing health care increased during this time [37, 38]. At the same time, maternal mortality decreased slightly between 2010 and 2015/2016, but remains a notable problem in Tanzania [37, 38].

**Table 1:** Summary of Decision Making and Access to Health Care Indicators from Demographic and Health Surveys

		Participation in decision making about a woman’s own health care (% of currently married or in union women age 15–49)			Person who decides how wife’s cash earnings are used (% of currently married or in union women age 15–49 who received cash earnings for employment in the 12 months preceding the survey)			Women’s problems accessing health care for themselves when they are sick (% of women age 15–49)		
Country	Year	Mainly Husband	Wife and husband jointly	Mainly wife	Mainly Husband	Wife and husband jointly	Mainly wife	Getting perm-ission to go for treatment	Getting money for treatment	Not wanting to go alone
Tanzania [37]	2010	38.1	45	15.3	16.6	47.2	35.9	2.4	24.1	10.5
Tanzania [38]	2015/ 2016	27.5	56.4	15.7	8.6	55.3	36.1	14.3	49.5	29.9
Burundi [39]	2010	22.6	63.6	13.6	12.6	65.1	22.0	37.3	77.0	41.1
Kenya [39]	2014	20.9	40.1	38.6	8.7	41.2	49.4	6.0	36.7	10.6
Rwanda [39]	2014/ 2015	16.0	60.1	23.2	12.4	67.5	19.5	2.7	49.3	17.6
Uganda [39]	2011	39.1	36.9	23.3	14.3	30.9	52.7	5.5	48.8	22.4

Tanzania reports similar decision-making patterns as other countries in the East African Community including Burundi, Kenya, Rwanda, and Uganda, although in Kenya and Uganda about half of women report deciding how their own earnings are used compared to a little over one third of women in Tanzania (see Table 1) [37–39]. Barriers to accessing health services are also ubiquitous in these countries, with the problem of getting money for treatment cited by the highest percentage of women in each country [37, 38].

Although government policy stipulates the provision of free maternal and child health services in Tanzania, the policy has been difficult to implement [40]. A study conducted in western Tanzania found that among women who delivered at a public facility, 62.5% paid for services, with an average cost for delivery of 3840 Tanzania Shillings (TZS) (USD3.08 at the time of the study) [41]. This cost increased to 6268 TZS (USD5.03 at the time of the study) when components of health expenses including transportation, drugs, and supplies were considered [41].

### The maternal and newborn health intervention

This study was part of a larger evaluation of an integrated maternal and newborn healthcare program implemented in partnership between the Ministry of Health and Social Welfare (MoHSW) and Jhpiego. The program sought to increase facility-based care during pregnancy and delivery in Morogoro Region, Tanzania. Further program details are available from Bishanga et al. (2018) [42] and Lefevre et al. (2015) [43].

### Sample

Study participants were male respondents who resided in one of the five focus districts of Morogoro Region and were partners of women who met the study criteria of giving birth within the previous 14 months and not experiencing complications during delivery. The researchers purposefully selected the 14-month period to reduce recall bias among study respondents and to allow time for outcomes measured as part of the larger study, such as re-initiation of contraceptive use among women [44] and use of postnatal care services [45]. The research excluded men whose partners experienced complications during delivery in order to avoid skewing the data toward more intensive health service requirements beyond routine childbirth. The sample included men whose partners delivered both at health facilities and in the community. Researchers selected respondents from villages near (< 3 km) and far (≥3 km) from health centers, as identified by facility-based staff.

Twenty-seven men meeting these criteria provided informed consent before being interviewed. The ethical review boards of Johns Hopkins University School of Public Health in Baltimore, USA and Muhimbili University of Health and Allied Sciences in Dar-es-Salaam, Tanzania provided ethical approval for this study.

### Data collection

Research assistants (RAs) received 5 days of training on maternal and newborn health, qualitative methods, and research ethics, followed by piloting and tool revision. The RAs, all bilingual Swahili and English speakers, included male and female social science graduate students and teachers.

Strategies to collect trustworthy data followed the approach of naturalistic inquiry put forth by Lincoln and Guba (1985), including prolonged engagement and analyst triangulation (see Table 2) [46]. The RAs conducted semi-structured in-depth interviews with the 27 men over a period of 2 months. Interview guides probed on experiences of care seeking during pregnancy, delivery, and the postpartum period, as well as how men view their role in maternal and child health. RAs and respondents were gender-matched to diminish gender-related influences that could affect data quality. The data collection phase included daily debriefing sessions amongst the research team [47]. Regular discussion during these debriefings provided opportunities to facilitate analyst triangulation, identify when to seek follow-up interviews, and iteratively refine data collection tools. Debriefings resulted in the decision to conduct follow-up interviews with three men in order to gain clarity and to further probe on responses insufficiently addressed in earlier interviews. All interviews ranged in length from 35 to 99 min, with an average length of 69 min. Each interview was digitally recorded, checked for quality, and transcribed verbatim in Swahili.

**Table 2:** Study rigor as informed by Lincoln and Guba (1985) and McMahon and Winch (2018)

Principle^a^	As Enacted in this Study
1. Prolonged engagement	- The research team was living in this setting and actively working on the larger evaluation for a minimum of six months, but more often several years. Via this immersion, the research team was attuned to the behaviors, priorities, and relationships inherent to this study setting.
2. Analyst triangulation	- Debriefings^b^ were conducted each night throughout data collection and involved all members of the data collection team sharing, comparing, amplifying, or refuting one another’s findings. Findings from debriefings were presented to Tanzania-based researchers, program implementers, and policymakers engaged in maternal health programs for feedback. Debriefing memos formed the basis for an audit trail of the study. - Members of the research team who undertook data collection also participated in data analysis. During analysis, at least two analysts analyzed each theme report and compared interpretations. - Throughout analysis, in the event of discrepancies, two senior researchers who were present throughout data collection and led most debriefings, weighed in and determined a way forward (highlighting opportunities for re-translation of interviews as necessary) - All final results were reviewed by the full research team (including most data collectors)

^a^As informed by Lincoln and Guba 1985 [41]; ^b^As informed by McMahon & Winch 2018 [42]

### Data analysis

Data analysis followed a modified framework approach [48]. Analysis started in the field during debriefings with the research team, when themes began to emerge [42]. Co-authors, during a data analysis workshop, further drew out themes from a selection of qualitatively rich transcripts – an inductive approach [49] – and later applied a questionnaire based on those themes a priori to all transcripts through a deductive approach [50]. Throughout the data analysis process, the authors returned to analyst triangulation to facilitate trustworthy conclusions from the data (see Table 2) [46].

To begin the inductive phase of analysis, the lead author and last author reviewed translated portions of transcripts identified during initial analysis conducted in ATLAS.ti, a qualitative data management software [51]. They chose three transcripts that were illustrative of the experiences of male respondents. Swahili-speaking co-authors (three of whom also participated in data collection) analyzed these transcripts in full in their original Swahili to retain their original context and meaning. The analysts and lead author then discussed themes that emerged from the coded transcripts to triangulate findings.

Analysts used Gulliford et al’s (2002) framework on access to health care [52], identified based on congruent themes, to inform the creation of a questionnaire deductively applied to all interview transcripts [50]. The lead author compiled the results of the questionnaire into theme reports. At least two analysts further analyzed each theme report to continue to triangulate findings and outline similarities and differences emerging across transcripts. Analysts then prepared summary reports including the translation of selected quotations into English. When translations differed between reports, an author re-translated the text. Routine dialogue took place among workshop participants to corroborate the findings across researchers [50].

## Results

### Study population

Table 3 summarizes demographic characteristics of the men interviewed. Men ranged in age from 22 years to 60 years, with a median age of 34 years. A majority of men were married (85%) and had, on average, three children. Most men had completed primary school (63%) and approximately half lived far from a health center. The majority of respondents’ partners gave birth to their most recent child at a health facility (82%), while a few delivered at home (7%) or en route to a health facility (11%).

**Table 3:** Demographic characteristics of respondents

Characteristic	Total (*n* = 27)	Percent (%)
Age and number of children		
Age (years) (Median/Range): 34/22–60 (*n* = 25)	N/A	N/A
Age 20–29	9	33.3
Age 30–39	8	29.6
Age 40–49	4	14.8
Age 50–59	3	11.1
Age 60–69	1	3.7
Unknown	2	7.4
Number of children (Mean/Range): 3.24/1–11 (n = 25)	N/A	N/A
District		
Kilombero	1	3.7
Kilosa and Gairo (one district at time of study)	10	37.0
Morogoro Rural	2	7.4
Mvomero	8	29.6
Ulaya	6	22.2
Distance from health center		
Near (< 3 km)	12	44.4
Far (> 3 km)	15	55.6
Marital status		
Married	23	85.2
Single	1	3.7
Engaged	1	3.7
Not reported	2	7.4
Level of education		
Started primary school	3	11.1
Completed primary school	17	63.0
Completed secondary school	3	11.1
No formal education	3	11.1
Not reported	1	3.7
Delivery location of most recent birth		
Health facility	22	81.5
Home	2	7.4
En route to facility (birth before arrival)	3	11.1

### The three delays

**Fig. 1:**
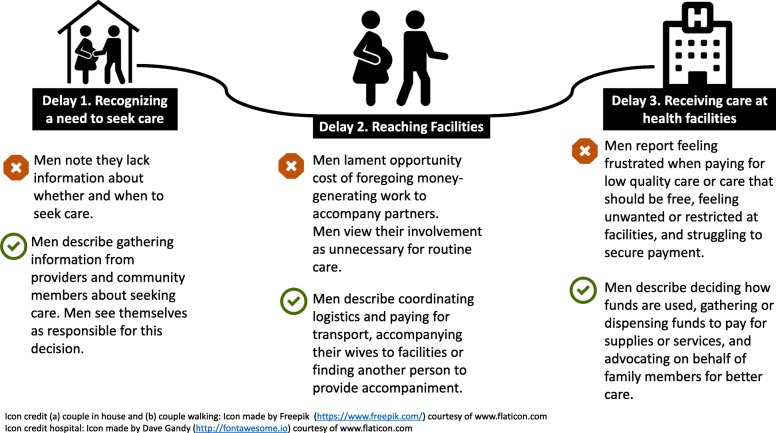
The Three Delays Model Applied to Male Involvement in Maternal Care Seeking

We found that the data collected from men align closely with the three delays model. We therefore present findings through the lens of this framework, highlighting how men described their roles in accessing care, and barriers to their involvement (see Fig. 1). While men did not describe their roles as uniformly positive, they generally highlighted the ways they try to be supportive partners for maternal care seeking and emphasized with depth, nuance, and conviction their efforts to facilitate access to care, particularly for non-routine care.

#### Delay 1: Men’s role in deciding to seek care

Men reported participating in the decision to seek care by using their role as decision maker positively, being supportive of facility-based care, and seeking advice from others. However, men cited a general lack of knowledge as a barrier to their involvement in deciding to seek care (see Fig. 1).

Male respondents referred to themselves as the “head of the household” or the “final decision maker” even when their partners were included in discussions, and indicated that they felt both proud of this role and also responsible for the health of their families upon becoming a husband or father:

*We used to discuss and agree together on healthcare seeking but I’m a final decision maker, even if she falls sick, because I took her from her parents so long ago. I’m responsible for that and in case things become more difficult for me I will let other people come and help me* (M23, age 40–49 years, 4 children, far from a health center).

Men described encouraging, even “insisting,” their partners seek care (“whether I am around or not”) and recognized facility-based care as “important” and a “right” because of the benefits of available services including vaccinations, testing for and treating diseases, counseling services, antenatal care, and the prevention of problems during delivery. Although respondents described feeling excited about new pregnancies, they also felt nervous and ill-informed in terms of how to “protect” their families and to “know what we are supposed to do, how to prepare ourselves and take care of our wives” (M10, age 20–29 years, 1 child, far from a health center).

Men reported seeking advice from community members, such as their parents who generally know more, “because we are still young and we may decide on something wrong” (M9, age 20–29 years, 1 child, near a health center). Men also described relying heavily on the expertise of facility-based providers, whom men usually held in high regard. For example, one respondent stated: “I can’t understand well because I am not a doctor, I can see some symptoms on my child but the doctor is the one who knows deeply” (M1, age 20–29 years, 1 child, far from a health center). Several men also described trusting the skills of facility-based providers over those of traditional birth attendants, which influenced their decisions about care seeking:

*During delivery the qualified traditional birth attendant was there but I was not ready to let her attend to my wife, so I decided to go directly to a hospital because she may cause a problem and leave [my partner] with scars or she may delay getting the child who will die due to my own fault* (M1, age 20–29 years, 1 child, far from a health center).

#### Delay 2: Men’s role in reaching care

The coordination of transportation logistics and the accompaniment of partners to facilities, especially for non-routine care, emerged as salient themes pertaining to men’s role in reaching care. However, men also discussed reasons for their lack of involvement in their partners reaching care, including the opportunity cost of accompaniment and the perception that accompaniment is not always essential for routine care (See Fig. 1).

Respondents described playing an active role in helping their partners reach care. They stated that they were expected to coordinate the necessary transportation logistics, especially in the event of childbirth (or other non-routine care), a referral, or “a serious issue.”

Geographic location emerged as a challenge in accessing services, especially during the rainy season when roads and river crossings are difficult or impossible to navigate and the cost of transportation increases. Men also described distance as a critical reason to accompany a pregnant woman (here referred to as “sick”) for care: “I am the one who took her there because it’s far away, six miles. A person can’t go on her own when she is sick. So I use my bicycle or we go by foot” (M23, age 40–49 years, 4 children, far from a health center). In addition, men accompanied their partners for HIV testing and to procure needed supplies or medications “so that I know how much it costs because medication is not found [at the health center]” (M5, age 30–39 years, 1 child, near a health center). However, even when men took responsibility for accompanying their partners, they revealed that a lack of advance preparation of funds and transportation can cause delays accessing care when it is urgently needed.

Compared to childbirth, unplanned care, or referral-level care, men described themselves as less inclined to accompany their partners for routine care, such as antenatal care, especially when the health facility was not far away. When making this decision, men reasoned that: 1) routine services rarely involved unforeseeable costs; 2) women were physically able to walk to care on their own (unlike during delivery or severe illness); and 3) women often attended these services with female companions.

Some men also said they could not accompany their partners to facilities due to the high opportunity cost of foregoing income-generating activities (e.g. farming, building, and preaching), but described making alternate arrangements for their partners, including paying for transportation, such as a bus, or finding someone else, such as a family member or a neighbor, to accompany their partner. However, men stated that sometimes women seek care on their own, as one respondent explained:

*I have many commitments, and above all I do not have the ability to escort her and come back to be able to do my work … she is used to going on her own very often* (M13, age 30–39 years, 1 child, near a health center).

#### Delay 3: Men’s role in ensuring care received at facilities

A lack of personal funds, restrictions on the presence of men at facilities, and a sense that facilities are inhospitable toward men are barriers to male involvement at the facility level. Nevertheless, men reported playing a large role in ensuring patients received care at facilities, including by paying for medications and supplies, acting as patient advocates, and petitioning for higher quality services (see Fig. 1).

As a primary role, men described their involvement at facilities as centering on the provision of funds to purchase medicines or supplies from nearby pharmacies. Men frequently noted that public facilities lack essential supplies. One man likened getting a diagnosis from a clinician to playing a game of “trial and error” because of a shortage of appropriate diagnostic supplies. Another man stated that his child was not able to get a vaccination, even after visiting the facility four times, due to stock outs of vaccines. As a result, men purchased needed items (medications being the most frequently discussed) so that their partners and children could receive care. Men lamented that pressure to provide necessary funds made them feel distressed and fearful for their family members’ lives:

*The doctor’s duty is just to write [a prescription]. Then you are told that there are no medicines; you are supposed to buy from pharmacies. This means that if you have money you will be treated and if you don’t have money [you will not be treated].* (M12 age 50–59 years, 7 children, near a health center).

Men also felt frustrated about paying for medications and supplies that they understood should be provided at no extra cost. Men described this frustration in three contexts: 1) making payments described at the health facility as being required to receive services, although government policy stipulates that certain populations (pregnant women and newborns) receive free care; 2) paying for medications and supplies that are stocked out at the facility in addition to payments described above—perceived as a form of double payment; and 3) paying for medications and supplies even after having contributed to a Community Health Fund (CHF), a form of community-based insurance covering a household’s health services. One man explained that people might decide to go to a higher level facility that is assumed to be better equipped but farther away instead of going to a nearby health center and “wasting your money because you will be told that there are no drugs” and then having to purchase them separately*.* Men also described how such frustrations may compel families to attend private facilities:

*We were told that children under five are supposed to get free services, but here in our place we don’t get those services because even for this one month old child I already bought medicine, that is why we are running away to avoid paying 1500 TZS (USD 0.95) and at the same time you won’t get medicine. So, it’s better if I go to a private hospital because there are no changes at the health center* (M4, age 30–39 years, 3 children, near a health center*).*

Along with frustration about paying for services and supplies that should be free, men described the cost of joining and maintaining memberships in CHFs, which have an annual fee of 5000 TZS (USD3.18) (this fee has since increased and in December 2017 was 10,000 TZS). Men described the futility of paying this annual fee as even those “who have paid that big amount of money suffer every day because there are no medicines at the facility” (M3, age 50–59 years, 7 children, far from a health center).

In response to poor service, men reported being outspoken about what they saw as unsatisfactory situations (e.g. shortages of staff, drugs, or supplies; rude, inattentive, or inexperienced providers; long wait times; and facilities being closed at night) and acting as patient advocates while at the facility, including by leveraging personal relationships. One man identified how he saw his role in finding adequate care for the patient:

*I am the head of my home. If I see that this treatment is going badly [at the dispensary], or not going well, then I say ‘hey’ or I tell the nurse or the doctor ‘my patient has been here for a week and I see that her health has not changed. Therefore I would like to try somewhere else.’ They allow you, and you go to [a referral facility].* (M3, age 50–59 years, 7 children, far from a health center).

Respondents fulfilled the role of patient advocate when, individually or with community members, they reported confronting authorities and health providers and petitioning for change. However, men described feeling disappointed, as “they don’t listen to us.” One man who explained that people have died while waiting to see a doctor joined others to report this problem to three levels of authority: the doctor, the in-charge at the facility, and the village government. In addition to advocating for general change, men also advocated on behalf of specific patients. The man whose child had missed vaccinations because of inadequate supply complained to the head of the health center. Another man tried negotiating at the health center on behalf of his pregnant wife to avoid paying both to receive health services and for out-of-pocket medication costs:

*[The staff at the health center] told my wife to give them 1500 TZS (USD0.95) … I went to see the doctor, and I told the doctor that I bought medication with the money that I had. Therefore, I do not have any money in my pockets. He said, ‘Now, what can we do?’ I said, ‘Now that is your responsibility. You can treat the patient, or you can [decide] not to treat her, because I have no money. I had it, but I have bought medication. If there were drugs here at the facility, it would be okay, I would have been able to pay the 1500 TZS. But I have to go and buy medication at the shop … How do you see that young man?’* (M5, age 30–39 years, 1 child, near a health center).

At health facilities, men also described feeling physically restricted. Men said they were not allowed or were not invited to be near their partners after “they took her” for labor and delivery. One respondent explained that he “will stay outside” while his partner’s mother was present during delivery. Another respondent reported that, despite movement restrictions, he remained peripherally engaged: “I couldn’t enter there because I am a man, so I inquired about the baby’s situation and was told that she was doing fine, and I went back and in the evening I communicated with them and they told me [my wife and child] were fine” (M21, age 20–29 years, 1 child, near a health center).

## Discussion

This study highlights men’s descriptions of their engagement along the three delays continuum, including filling the roles of decision maker, financial provider, and patient advocate. As decision makers, men appraise opportunities for their involvement and make financial decisions about care seeking. As financial providers, men evaluate the cost of care versus quality. As patient advocates, men navigate the health system on behalf of their families. We will examine each of these findings relative to the literature, followed by a discussion of the ways in which men’s insights can inform program implementers. We also highlight the importance of considering male-focused program interventions in the context of gender inequity.

Study respondents viewed themselves as accountable for the health of their families, and often highlighted their role as decision makers who draw on available (sometimes inadequate) information. Men described making decisions about their involvement based on the urgency of the situation and on the nature of care sought. They considered routine and non-routine care differently, the latter requiring a higher level of male involvement, which was also found by Kwambai et al. (2013) in Kenya [20]. Men interviewed also based decisions about their availability for involvement in care seeking on the opportunity cost of leaving income-generating activities. However, our findings show that, when men are not physically present, they nevertheless make essential decisions about care seeking. Similarly, in Ghana and Malawi men outlined plans for birth preparedness, coordinated payment for transportation, arranged financial support from afar, and appointed someone else to help their partner [8, 39].

Making difficult financial decisions in a context of limited resources emerged as a highly salient issue in this study. Despite challenges, men in Morogoro and across other low-resource settings described removing barriers to access by paying for health services [53–55], procuring or preparing food [16, 53, 55, 56], arranging and paying for transport to facilities [16, 19, 20, 53, 56], buying medications [16, 53, 55], and buying birth kits [57]. However, similar to men in studies in Ghana, Nepal, and Malawi, men in our study discussed struggling as they gathered and dispensed funds [8, 16, 19, 56]. A lack of funds, prohibitively expensive care, or inadequate financial preparations meant that men were not always able to fulfill the role of financial provider, a finding documented previously in Tanzania [7, 18]. Similar to sentiments reported from Malawi, men interviewed in Morogoro were painfully aware that a lack of funds could mean denial of care, and possible death, for a family member [56].

Findings from this study are consistent with others that highlight the challenges men face regarding the cost of healthcare expenditures and the question of cost relative to quality of care available [3–5, 41, 58]. As financial providers, men are frustrated when they perceive a lack of value in health spending. Respondents often voiced this frustration in relation to having to pay for medications and supplies on top of health service fees and CHF fees, particularly when they understood that a good or a service should be provided for free. This perception of not getting value for healthcare expenditures, especially related to drug and supply availability, can make people less likely to seek care at all, as described in Zambia [59], or can make men more likely to discourage their wives from using facility-based care, as described in Nigeria [54].

Dissatisfaction with quality, as appraised through the lens of financial decision making, presents an opportunity for men to also play the role of patient advocate. In our study, men described the need to navigate the health system to find locations that best met their expectations of optimal care. Similar findings in Tanzania presented by Kruk et al. (2008) found that frustration with stock outs at public facilities can drive people to seek care at more expensive but better equipped facilities [41]. These researchers reported that, of women delivering in a facility, almost half delivered in a mission facility to receive higher quality care, including better availability of supplies, despite having to pay on average four times more on direct medical costs (provider fees, diagnostic tests, supplies, and medications) [41].

In addition to navigating the health system to locate better quality care, men also help facilitate and advocate for needed care after arriving at facilities. Aarnio, Chipeta, and Kulmala (2013) found that men in Malawi may feel more purposeful during delivery at facilities where they provide financial and other support, compared to home births where they might be less occupied [56]. However, as described by Kaye et al. (2014) in Uganda and August et al. (2016) in Tanzania, men can still feel ineffective at the facility when their role is not clear [23, 26]. Men in our study reported taking action by going to pharmacies to buy supplies and medications and being outspoken about problems such as stock outs, long wait times, and quality of care. They tried to enact change by advocating for patients and registering complaints with community and health center officials, a role that can be challenging to assume as shown by men in Rwanda who did not feel empowered to challenge health facility norms and infrastructure limitations [21]. These findings point to the importance of the active role that male respondents filled when advocating for and facilitating effective care at facilities, especially when we consider that avoiding delays once at facilities is particularly important for reducing maternal deaths [60]. In fact, though researchers have previously tied male influence to the first two delays of the three delays model [56], our study explicitly ties the importance of men’s role to avoiding the third delay at the facility level.

### Respondent-driven recommendations

Our study reflects others in noting that men would benefit from and be receptive to more education about maternal health, including danger signs and how to prepare for delivery and complications [54, 56, 57]. This education can be delivered at community and facility levels. At the community level, promising interventions for educating men include peer education, community meetings, and the distribution of educational materials [5]. Facility-based educational opportunities include promoting and welcoming male involvement throughout the pregnancy, delivery, and postpartum periods and sharing health information with men when they encounter the health system during these care delivery touchpoints [5, 6, 21, 56, 57]. This promotion of male participation is particularly important considering that a lower level of male involvement in routine services, such as antenatal care (when health messages are often communicated), may be a contributing factor to the lack of health knowledge that men described [21, 57].

Interventions seeking to shift community norms regarding male participation are also recommended [18]. As shown by a study in Ghana, when men are seen at the community level as being supportive of facility-based deliveries, women are significantly more likely to deliver at a facility [61]. Sharing examples from studies like ours of the positive roles men can play in care seeking could thereby contribute to shifting community-level perceptions of male involvement.

At the facility level, our study and others from Ghana and Uganda show that clearly defining supportive roles for men at facilities is important [8, 23], as is creating male-friendly environments, a finding emerging from research in Ghana, Uganda and Tanzania [8, 23, 26]. However, for interventions that aim to increase facility-based care through male involvement to have the desired effect of improving maternal and newborn health, confidence in several institutions must be restored. Families need to see that care that is reportedly free is actually free and that savings schemes such as CHFs are honored. Most importantly, the care offered at facilities must be truly effective, affordable, accessible, and of high quality [54].

### Considerations of gender inequity

As a final point, programs implementers should consider interventions that focus on male involvement in the context of gender relations and inequalities. Men’s ability to more quickly address financial or transportation barriers through their control of resources is problematic [55, 62]. Moving childbirth from the home setting to the more resource-intensive, and therefore more male-dominant, setting of the health facility also has implications for the agency of women during childbirth [19, 36]. Furthermore, advocating for male involvement can adversely affect women who do not have or are unable to involve their partners because their partners refuse to participate, do not work locally, are abusive, or have passed away. For example, enforcing shorter wait times for couples unfairly disadvantages single women and devalues women’s time compared to men’s [19, 20, 22]. Even when husbands want to be involved, requirements for their attendance could delay their partners’ receipt of care [21]. Lastly, women may not want their husbands present during some health services, including physical examinations, delivery, or discussions about sexually transmitted infections [5, 63].

For these reasons, male involvement should be encouraged alongside efforts to promote women’s autonomy and inter-spousal communication [16]. Interventions that have not adequately taken gender relations into account have seen negative effects on maternal health care seeking [26]. It is therefore important that programs engage both men and women in designing and providing feedback about interventions, as well as piloting and revising these interventions as needed [5].

### Study limitations

Response bias and the translation process may limit this study. Social desirability bias could affect our data if respondents provided answers to questions that they thought would be favored by the interviewer. Recall bias could also affect responses when men described events or conversations that occurred in the past. Through probing and rapport building we sought to diminish these biases. In addition, RAs conducted all interviews in Swahili, which were then transcribed, and select quotations translated into English. Though an author re-translated text if discrepancies in translation occurred, it is possible that some nuances were lost during the translation process.

## Conclusions

Presenting data from men according to the three delays model highlights men’s roles in seeking facility-based care. Although there is still a need to improve how and how often men engage in maternal and newborn health, program implementers can learn from what men are already doing to create a role for themselves within a system that does not always facilitate their involvement and where the cost of accessing care is a struggle. Despite many barriers, men describe navigating the health system on behalf of their partners and children. They support facility-based care by arranging transport and accompanying their partners, purchasing supplies and medications that are not available at facilities, and advocating for improved care. Through these actions, men address some of the limitations in the public system that can prevent women from accessing care as quickly or as successfully on their own, limitations that must be addressed through health system strengthening and improved gender equity. These insights from men can inform future research and interventions that encourage men to be positively involved in maternal and newborn health.

## Data Availability

The interviews transcribed for the current study are not publicly available due to individual privacy considerations. Potentially following approval by the ethical review boards of Muhimbili and Johns Hopkins universities, the corresponding author could make transcripts available upon reasonable request.
